# Hospital Reform : A Reverie

**Published:** 1898-01-29

**Authors:** 


### HOSPITAL REFORM: A REVERIE.

The Hospital Reform Association may not be a very important body, but amid so much that is nebulous it has at least this advantage, that it has a definite programme, viz., that the medical profession should hold the key to the hospitals. At the meeting held last week Mr. Garrett Horder said the association had urged the necessity of restricting all out-patients to those bringing a recommendation from a medical man, this, in their judgment, being the best, easiest, and simplest way of limiting hospital treatment to those who were in need of it. This opens out many vistas of thought, many possibilities of action, and many new relationships between the medical profession and the public. Thinking the matter over we wondered how far those who subscribe the money to support these hospitals would care to hand over to the doctors the gentle pleasure of distributing their charity; we wondered how far the patients would care to ask their doctors, to whom they would often very likely be in debt, for tickets for the hospitals; we wondered whether the patient under such circumstances would really go to his own doctor, or would slip round to some one else; we wondered, again, if that were done, how much more this new doctor would know about the patient than an inquiry officer would; our fancy led us to imagine what the medical profession as a whole would do with this new honour, or this new labour, according to the point of view from which we choose to look at it, whether they would care for it, how they would use it, how they would abuse it, and what would then be the relations between the hospitals, the public, and the profession. This proved so endless and entangling a problem that weariness and sleep o'ercame us, and, as we slept, we dreamed of surgeries and consulting-rooms, of overworked practitioners worried by applicants for tickets, and of many other things besides. It was in a little surgery. The doctor, weary after his day's round, was faced by all the dispensing which he had to do—his dinner was waiting, and, if he was not quick, his evening patients would be upon him before it could be eaten—when there entered a person whom he had never seen before, not badly dressed, apparently a new patient, and respectable. The struggle of life is keen, new patients in some districts are by no means plentiful, and we need hardly be surprised that the dispensing was put aside, the dinner was delayed, and the patient was invited to unfold her tale of woe. In a consulting-room, inquiries as to a patient's ailments go on in a smooth and progressive manner; there are no breaks, no intervals at which to change the conversation; the patient passes from confidence to confidence, and it seems but a kindly interest in all that has been said that leads the doctor to make a physical examination. So naturally, in fact, does one thing lead to another, that this weary doctor had brightened up in his interest in his new case, he had listened to all the long history, he had begun to examine the patient, when she at last said: "But, you know, doctor, I'm not a patient; I only came to ask for a ticket for the hospital. I've always been under Dr. Jones, but I did not like to ask him, for, you see, we owe him a bit of money, and I'm expecting again in a little time, so I thought perhaps you would give me a recommend." And, if we remember our dream aright, the ticket was given. But as the poor doctor got his dinner, all gone cold, he did not mince his words in denouncing the new system to his wife. "It is monstrous; what do I know about the wretched woman? I know she's ill, but I know no more than the man in the moon that she is a proper case for a hospital." She: "I know I would have turned her out pretty quickly if I had known how your time was being wasted while your dinner was getting cold." He: "My dear, you do not know. There is Smith round the corner would do it in a minute, and Robinson up the next street would not only do it but would call to-morrow to ask how she got on, and very likely get engaged by her for taking such interest in her case, and I've no doubt even Jones himself would give her a ticket rather than lose them as patients. I know now who she is; they keep a little butcher's shop in T—— Street, and they are no more fit to be hospital patients than I am. But what can I do? What is to become of the practice if I get a name for being hard to the poor?" She: "Horrid wretch! Why those are the very people that would not leave the leg of mutton that I bought unless I gave the boy the money."

And then I dreamed of the really deserving cases—poor and worn down, full of troubles but short of clothes, knocking at door after door in Harley Street. "Please, Sir, I've come to ask the doctor for a ticket for the hospital." But what said the butler? Did he open the door and show that poor broken down specimen into the waiting room, to sit diffusing an odour of poverty, to dog-ear the illustrated papers, and to lead the doctor's comfortable patients to say that they had been "quite deceived as to the style of practice which he had?" Not at all. The butler merely said—"No, my good woman, he is busy, you must go to some-one else;" and if a word of protest was made the door was shut, with or without the adjunct "off you go"; and so on down the street, and through many other streets, till at last the sixpenny dispensary is arrived at. There, at least, poor and unknown as she may be, she is civilly dealt with, for she knows the ways, and pays her sixpence—and when she has done her talk, she simply says, "And I want a ticket for the hospital," for all the world as if she wanted half a pound of cheese. "Oh, certainly," he says, and gives it; and there the matter ends.

And then my dreams carried me to a smug consulting-room, where a sleek physician was opening his morning letters. "Lady —— presents her compliments to Dr. ——, and would be much obliged if he would send her a hospital ticket for her coachman's little boy, John Tivy, aged six, a most deserving case." And the bell was rung, and the butler brought a book of printed forms, and one of them he signed, enclosing it in a little note, saying he was "so glad to be of use;" and again the thing was done; and, of course, the benefits of hospital treatment were in this way secured to the deserving poor alone. But my dream had become a nightmare, and I woke, exclaiming, "Let the hospitals do their own work, and inquire into the circumstances of their own patients!"

